# Mutant-Allele Tumor Heterogeneity, a Favorable Biomarker to Assess Intra-Tumor Heterogeneity, in Advanced Lung Adenocarcinoma

**DOI:** 10.3389/fonc.2022.888951

**Published:** 2022-07-01

**Authors:** Xiaoxuan Wu, Peng Song, Lei Guo, Jianming Ying, Wenbin Li

**Affiliations:** ^1^ Department of Pathology, National Cancer Center/National Clinical Research Center for Cancer/Cancer Hospital, Chinese Academy of Medical Sciences and Peking Union Medical College, Beijing, China; ^2^ Department of Thoracic Surgery, National Cancer Center/National Clinical Research Center for Cancer/Cancer Hospital, Chinese Academy of Medical Sciences and Peking Union Medical College, Beijing, China

**Keywords:** intra-tumor heterogeneity, TP53, tumor mutational burden, mutant-allele tumor heterogeneity, lung adenocarcinoma

## Abstract

**Background:**

Intra-tumor heterogeneity (ITH) plays a vital role in drug resistance and recurrence of lung cancer. We used a mutant-allele tumor heterogeneity (MATH) algorithm to assess ITH and investigated its association with clinical and molecular features in advanced lung adenocarcinoma.

**Methods:**

Tissues from 63 patients with advanced lung adenocarcinoma were analyzed by next-generation sequencing (NGS) using a panel targeting 520 cancer-relevant genes. We calculated the MATH values from NGS data and further investigated their correlation with clinical and molecular characteristics.

**Results:**

Among the 63 patients with advanced lung adenocarcinoma, the median value of MATH was 33.06. Patients with *EGFR* mutation had higher level of MATH score than those with wild-type *EGFR* status (*P* = 0.008). Patients with stage IV disease showed a trend to have a higher MATH score than those with stage III (*P* = 0.052). MATH was higher in patients with disruptive *TP53* mutations than in those with non-disruptive mutations (*P* = 0.036) or wild-type sequence (*P* = 0.023), but did not differ between tumors with non-disruptive mutations and wild-type *TP53* (*P* = 0.867). High MATH is associated with mutations in mismatch repair (MMR) pathway (*P* = 0.026) and base excision repair (BER) pathway (*P* = 0.008). In addition, MATH was found to have a positive correlation with tumor mutational burden (TMB) (Spearman ρ = 0.354; *P* = 0.004). In 26 patients harboring EGFR mutation treated with first generation EGFR TKI as single-agent therapy, the objective response rate was higher in the Low-MATH group than in the High-MATH group (75% vs. 21%; *P* = 0.016) and Low-MATH group showed a significantly longer progression-free survival than High-MATH group (median PFS: 13.7 months vs. 10.1 months; *P* = 0.024).

**Conclusions:**

For patients with advanced lung adenocarcinoma, MATH may serve as a clinically practical biomarker to assess intratumor heterogeneity.

## Introduction

Lung cancer remains the leading cause of cancer-related mortality worldwide (1). Despite remarkable progresses have been made during the last decade, prognosis of patients with this disease is still disappointing. A pivotal factor leading to the lethal outcome, therapeutic failure, and drug resistance of lung cancer is intratumor heterogeneity (ITH) (2). Previous studies showed that high immune-ITH are predictive for worse clinical outcome in HCC patients, coexisting subpopulations of cells with heterogeneous gene expression leading to multiple, concurrent resistance mechanisms in SCLC, UVB-induced tumor heterogeneity impacts immune response in melanoma (3–6). Intratumor CMS heterogeneity was associated with worse prognosis in localized colon cancer (5). Hence, measurement of ITH may provide clinically significant information for treatment and prognosis prediction of lung cancer.

Multiregional biopsies and single cell sequencing were the conventional methods to detect and evaluate ITH (7–9), whereas these methods are too time consuming and labor intensive to be clinically applied on large population. In mixed tumor DNA, high mutation frequencies were observed in the ancestral clones and low mutation frequencies in subclones. Therefore, greater variability in mutant-allele fractions tends to be found in a genetically more heterogeneous tumor. Recently, calculated by the width to the center of distribution of mutant-allele fractions based on next generation sequencing (NGS) data, a novel algorithm has been proposed to measure genetic heterogeneity—mutant-allele tumor heterogeneity (MATH). Avoiding the practical difficulties of the conventional methods, MATH measures ITH directly using NGS data, which has been proven to be a simple yet effective way to assess ITH (10).

**Figure 1:**
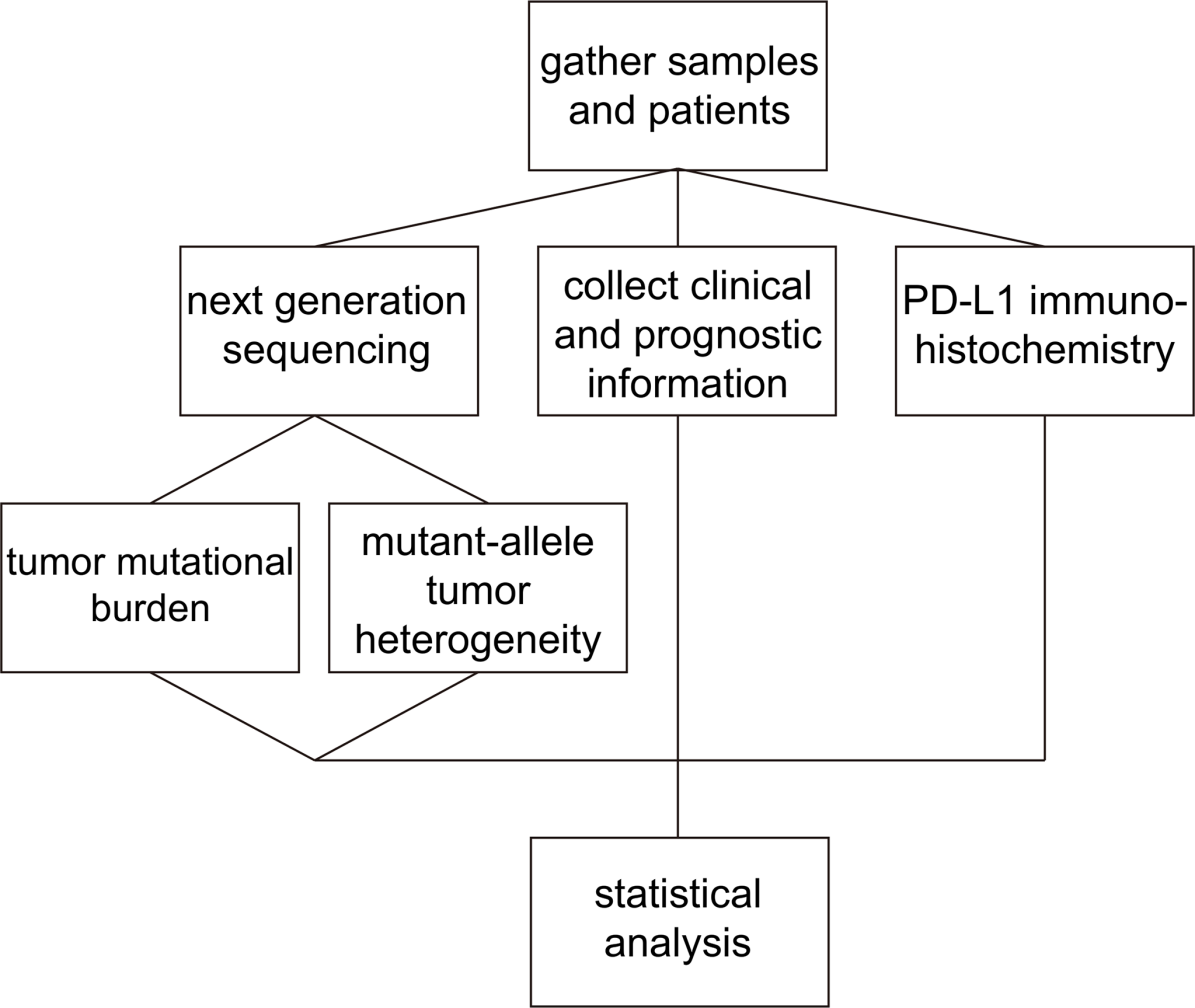
Schematic of the experiment and analysis process. Firstly, we gathered patients and samples and collected clinical and prognosis information. We conducted NGS to calculate MATH and TMB value for patients and performed immunohistochemistry analysis to identify PD-L1 expression status. Then, statistical analysis was conducted to explore the predictive value of MATH in advanced lung adenocarcinoma.

Intra-tumor genetic heterogeneity assessd by MATH was found to be significantly associated with overall survival in head and neck squamous cell carcinoma (11). And heterogeneity quantified by MATH score was correlated with the risk of metastases in colon cancer (12). Moreover, MATH score was proved to be a prognostic factor in male colorectal cancer patients (13). Although several previous studies had investigated the association of MATH with clinicopathologic features and prognostic value in colorectal cancer, breast cancer and head and neck squamous cell carcinoma, the clinical and molecular relevance of MATH in lung adenocarcinoma was still unknown (11, 13, 14). To explore the prognostic value of MATH in advanced lung adenocarcinoma, we first gathered samples and patients from four institutions and collected their clinical and prognosis information. Then, we conducted NGS to calculate MATH and TMB value for patients and performed immunohistochemistry analysis to explore PD-L1 expression status in tumor tissues from formalin-fixed, paraffin-embedded blocks. Based on the patients information and experiment data, we conducted statistical analysis to identify the clinical and molecular relevance of MATH in advanced lung adenocarcinoma as shown in **Figure 1**.

## Materials and Methods

### Patients and Samples

This retrospective cohort study included patients from four institutions: National Cancer Center/Cancer Hospital, Fudan University Shanghai Cancer Center, West China Hospital, Hebei Medical University Fourth Affiliated Hospital. A total of 63 patients diagnosed with advanced lung adenocarcinoma between January 2017 to June 2017 were enrolled in this study. Clinical and molecular data were collected, including age, sex, histological type, serum carcinoembryonic antigen (CEA), tumor status, lymph node status, distant metastasis, clinical stage, genomic alterations and PD-L1 expression. Patients in the cohort are clinically matched according to age and sex for better reliability. The tumor specimens used were formalin-fixed paraffin-embedded (FFPE) tissue specimens from archival tissue samples. This study was approved by our institutional review board and ethics committee of Cancer Hospital, Chinese Academy of Medical Science.

### Next-Generation Sequencing

The specimens of the 63 patients were obtained by aspiration biopsy and passed the NGS quality control procedure. Targeted NGS was performed as previously reported (15). In brief, genomic DNA was profiled by using a capture-based 520 cancer-gene panel (Burning Rock Biotech, Guangzhou, China). DNA concentration was measured with the Qubit dsDNA assay. Fragments of 200 to 400-bp sizes were selected with beads (Agencourt AMPure XP kit; Beckman-Coulter, Brea, CA), followed by hybridization with the capture probes baits, hybrid selection with magnetic beads, and PCR amplification. A bioanalyzer high-sensitivity DNA assay was then used to assess the quality and size range. Available indexed samples were then sequenced on Nextseq 550 (Illumina, San Diego, CA) for base substitutions, indels, CNA and DNA rearrangement analysis.

### Tumor Mutational Burden

TMB was determined as the number of base substitutions and indel mutations in the somatic coding region per megabase (Mb) of genome examined. Both synonymous and nonsynonymous alterations in the coding region of targeted genes were counted. Noncoding alterations were excluded and alterations predicted to be germline by the somatic-germline zygosity algorithm were not counted (16). Somatic alterations listed in the Catalogue of Somatic Mutations in Cancer (COSMIC) and truncations in tumor suppressor genes were not counted (17).

### MATH

The MATH value for each tumor was based on the distribution of mutant-allele fractions among tumor specific mutated loci. The procedures in determining the MATH value of an individual tumor from the NGS data were as follows (1): calculating the mutant allele frequencies (MAF) at each loci as the ratio of number of sequencing reads with mutant allele to total read depth. Due to lack of gene sequencing of normal germline samples, we only include heterozygous variants with MAF between 0.05 and 0.75 according to a previous report (12) (2). calculating the median absolute deviation (MAD): multiply a factor of 1.4826 by the median of the absolute differences of each MAF from the median MAF value (3). the MATH value for each tumor was defined as the percentage ratio of the MAD to the median of the MAFs: MATH = 100 × MAD/median. MATH was stratified by median in our cohort: low MATH (≤ 33.06) and high MATH (> 33.06).

### PD-L1 Immunohistochemistry

PD-L1 was the most widely used and efficient biomarker to predict immunotherapy in clinical practice. Immunohistochemical analysis of PD-L1 expression was performed as previously reported (18). In brief, tissue sections (4 mm thick) were cut from formalin-fixed, paraffin-embedded blocks containing representative tumors and processed for PD-L1 IHC. The presence of at least 100 viable tumor cells (TC) was required for the specimen to be considered adequate for quantification of PD-L1 expression. The expression of PD-L1 was evaluated by IHC staining using Dako 22C3 monoclonal antibody. PD-L1 IHC 22C3 pharmDx was performed on the DAKO Autostainer Link 48. TC showing either partial or complete cell membrane staining for PD-L1 were evaluated as positive cells. Tumor proportion score (TPS) was used to evaluate PD-L1 expression on TC, which was the percentage of PD-L1 positive TC showing partial or complete membrane staining in the overall tumor sections. PD-L1 expression on tumor-infiltrating immune cells (IC) was assessed as the proportion of tumor area occupied by PD-L1 positive immune cells of any intensity.

### Statistical Analysis

Patients and clinical and molecular characteristics were examined using the one-way analysis of variance (ANOVA) for qualitative variables with pairwise comparison adjusted using the Bonferroni test and the Mann–Whitney test for continuous variables. Correlations were estimated with the Spearman’s rank correlation coefficient. The survival curves were calculated by the Kaplan-Meier method and differences in survival were tested by the log-rank test. Statistical analysis was performed using SPSS version 20.0 software (IBM, Armonk, NY, USA). All P values were two-sided, and a P value of < 0.05 was considered statistically significant.

## Results

### Correlation Between Clinical Characteristics and MATH

**Figure 2:**
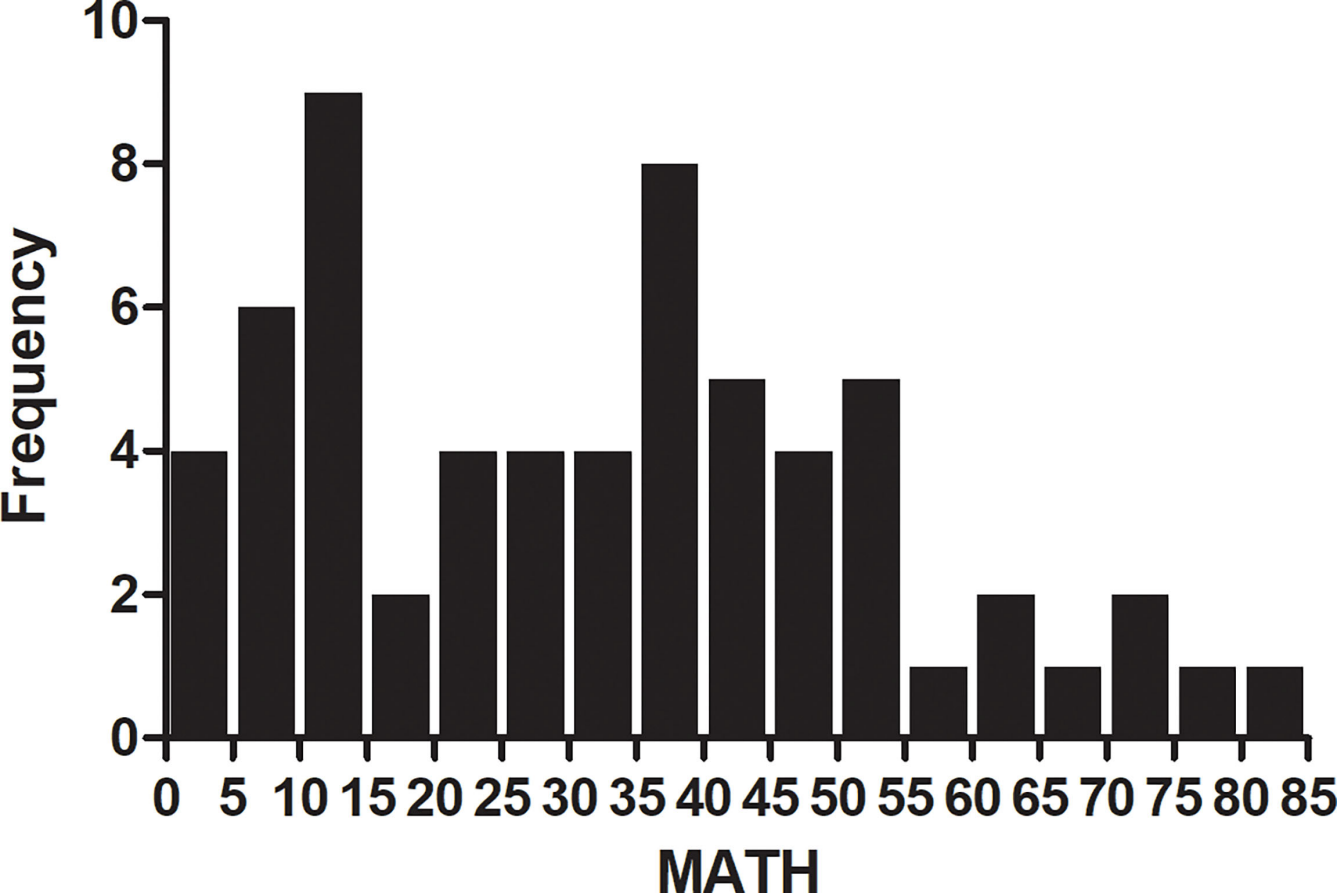
Frequency of mutant-allele tumor heterogeneity (MATH) score among 63 patients with lung adenocarcinoma. Patients with MATH 10-15 and 35-40 account for 9% and 8% portion. The median value of MATH was 33.06.

MATH with different clinical characteristics were analyzed by ANOVA in **Table 1**. All the 63 patients had a pathological diagnosis of lung adenocarcinoma and the majority had metastatic disease (87.3%). The mutation frequency of *EGFR* and *KRAS* was 50.8% and 19.0%, respectively. More than half of them (61.9%) were PD-L1 negative and only 14.3% were PD-L1 high expression (PD-L1 TPS ≥ 50%). The median value of MATH was 33.06. The distribution of MATH was shown in **Figure 2**.

**Table 1:** Association of clinical characteristics with MATH.

Characteristics	No. (% of Total)	Relation to MATH	
		MATH ± SD	*P*§
**Sex**			0.813
Male	32 (50.8%)	31.73 **±** 20.83	
Female	31 (49.2%)	33.00 **±** 21.53	
**Age** (years)			0.575
< 60	32 (50.8%)	30.85 ± 19.01	
≥ 60	31 (49.2%)	33.85 ± 23.02	
**Histology**			NA
ADC	63 (100%)	32.19 ± 21.42	
Non-ADC	0 (0%)	0	
**Tumor location**			0.142
Right	41 (65.1%)	35.23 ± 21.28	
Left	22 (34.9%)	27.05 ± 19.93	
**CEA**			0.532
Normal	5 (7.9%)	42.63 ± 33.57	
Elevated	18 (28.6%)	31.56 ± 18.44	
NA	41 (65.1%)	31.46 ± 20.60	
**T classification**			0.112
1	10 (15.9%)	38.12 ± 20.74	
2	25 (39.7%)	29.55 ± 21.58	
3	18 (28.5%)	39.29 ± 19.88	
4	10 (15.9%)	21.24 ± 18.30	
**N classification**			0.664
0	5 (7.9%)	27.06 ± 25.31	
1	4 (6.4%)	24.85 ± 12.09	
2	24 (38.1%)	30.83 ± 21.81	
3	30 (47.6%)	35.50 ± 20.97	
**Clinical stage**			0.052
III	8 (12.7%)	17.86 ± 12.28	
IV	55 (87.3%)	34.19 ± 21.25	
**No. of metastatic sites**			0.066
0	8 (12.7%)	17.86 ± 12.28	
1	29 (46.0%)	26.97 ± 19.40	
≥ 2	26 (41.3%)	35.95 ± 21.51	
**Brain metastases at Dx**			0.906
Yes	15 (23.8%)	32.55 ± 20.34	
No	48 (76.2%)	31.81 ± 23.84	

ADC, adenocarcinoma; CEA, carcinoembryonic antigen; MATH, mutant-allele tumor heterogeneity; NA, not available.

**§**By analysis of variance (ANOVA).

We examined the association between tumor MATH values and clinical variables. In one-way analysis of variance (ANOVA), patients with stage IV tend to have a higher MATH score than those with stage III, although the difference was not statistically significant (*P* = 0.052). No other clinical characteristics was found to have significant association with MATH.

### Correlation of MATH With Common Driver Mutations of Lung Adenocarcinoma

Among all 63 patients, 32 patients harbor *EGFR* mutation, 12 *KRAS* mutation, 5 *ALK* rearrangement, 2 *ROS1* rearrangement, 4 *MET* amplification, 1 *RET* rearrangement and 2 *HER2* mutation. Twenty-nine of 32 (90.6%) patients with TMB-High had one of the common driver mutations, whereas 22 of 31 (71.0%) patients with MATH-Low had one of the driver mutations. The presence of driver mutation in EGFR was associated with higher MATH score (P = 0.010), whereas KRAS mutation had no significant correlation with MATH score (P = 0.707). Additionally, only one of 5 patients with ALK rearrangement had high MATH score and 2 of 4 patients with *MET* amplification had high MATH score. Two patients with *ROS1* rearrangement both had low MATH score and 2 patients with *HER2* mutation both have high MATH score. Moreover, one patient with *RET* rearrangement had high MATH score.

### Association of MATH With *TP53* Mutations and DNA Repair Pathway Mutations

**Figure 3:**
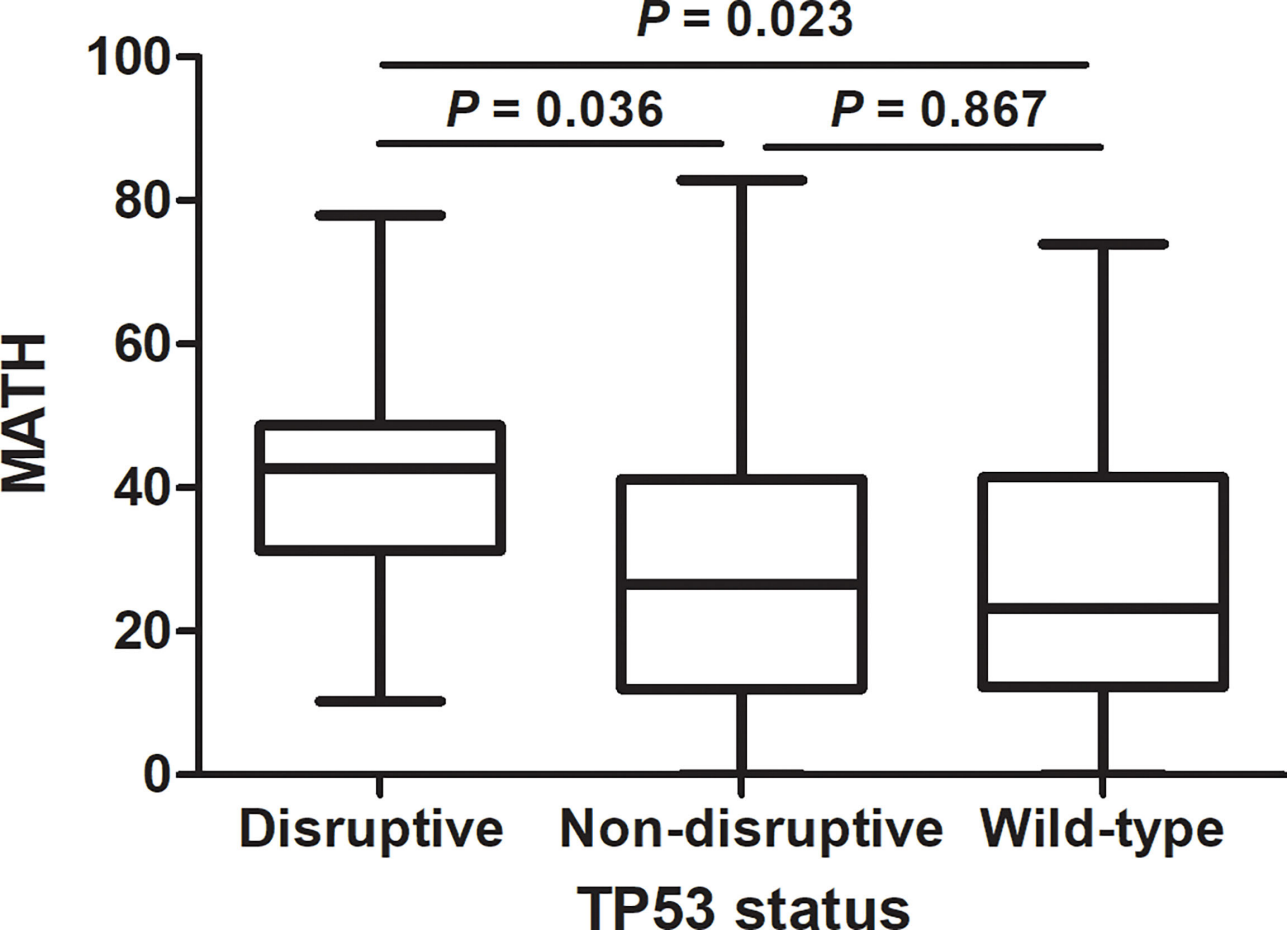
Relation of mutant-allele tumor heterogeneity (MATH) to *TP53* mutation status in lung adenocarcinoma. MATH was higher in patients having disruptive TP53 mutations than in those with non-disruptive mutations (P = 0.036) or wild-type sequence (P = 0.023).

As previously reported (19), we classified *TP53* mutations into two types, disruptive and nondisruptive, and observed 15 disruptive mutations and 20 nondisruptive mutations. MATH was higher in patients having disruptive *TP53* mutations than in those with non-disruptive mutations (*P* = 0.036) or wild-type sequence (*P* = 0.023), but did not differ between tumors having non-disruptive mutations and wild-type *TP53* (*P* = 0.867) (**Figure 3**).

**Figure 4:**
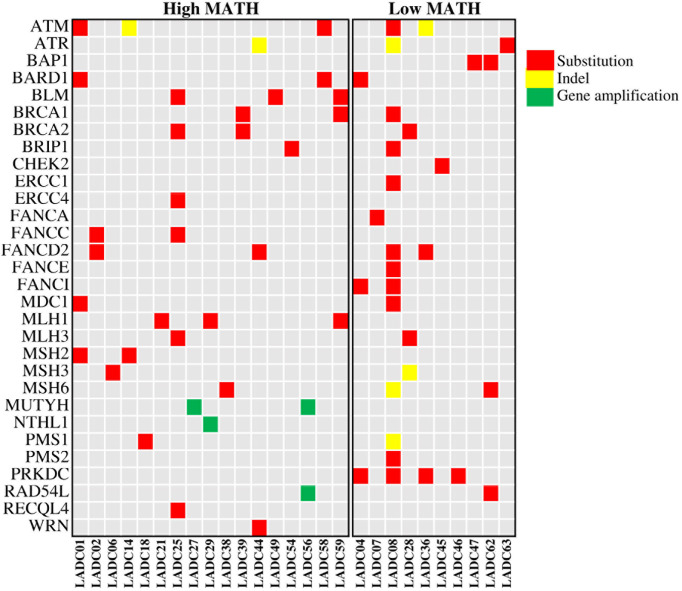
Heatmap showing genomic alternations of genes in DNA repair pathway in 27 LADC samples. LADC indicates lung adenocarcinoma. A total of 27 patients have DNA repair pathway mutations, including 24 mutations in HR pathway, 15 mutations in MMR pathway, 3 mutations in BER pathway, 2 mutations in NER pathway and 4 mutations in NHEJ pathway.

**Figure 5:**
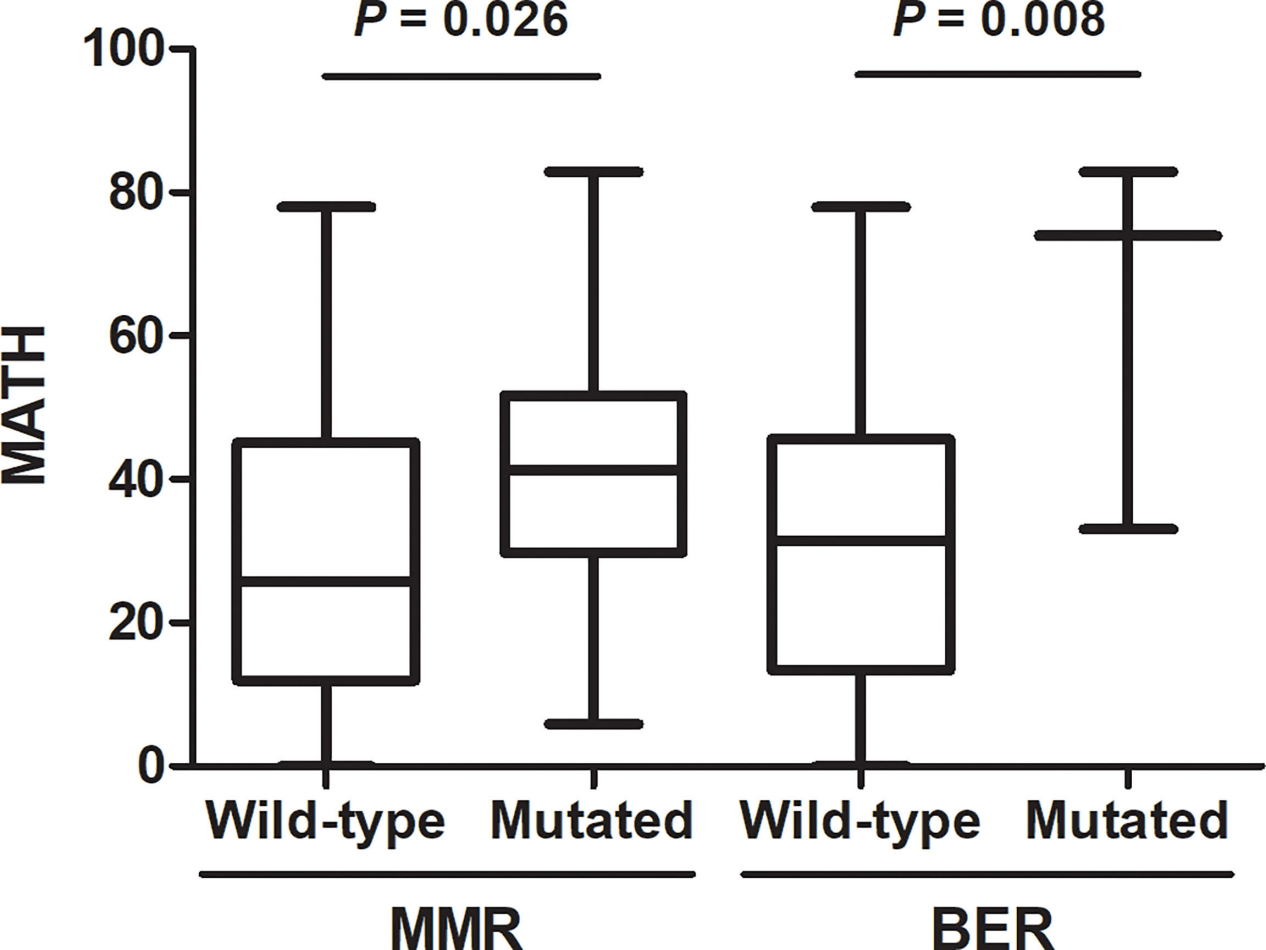
Association of DNA repair pathway mutations with mutant-allele tumor heterogeneity (MATH). Presence of non-silent somatic mutations in mismatch repair (MMR) (P = 0.026) or base excision repair (BER) (P = 0.008) were associated with high MATH.

We then checked the presence of non-silent mutations in the DNA repair pathways. Mutations occurred in genes of DNA repair pathway are main contributors to the somatic mutations in tumor. Specifically, we looked at the frequency of mutations in homologous recombination (HR), mismatch repair (MMR), base excision repair (BER), nucleotide excision repair (NER), and nonhomologous end joining (NHEJ) pathways (The genes involved in these pathways are listed in **Supplementary Table 1**). A total of 27 patients have DNA repair pathway mutations, including 24 mutations in HR pathway, 15 mutations in MMR pathway, 3 mutations in BER pathway, 2 mutations in NER pathway and 4 mutations in NHEJ pathway. Genomic alternations of genes in DNA repair pathway were shown in **Figure 4**. We found that high MATH was associated with mutations in mismatch repair (MMR) pathway (P = 0.026) and base excision repair (BER) pathway (P = 0.008) (**Figure 5**).

### TMB, MATH and PD-L1 Expression

**Figure 6:**
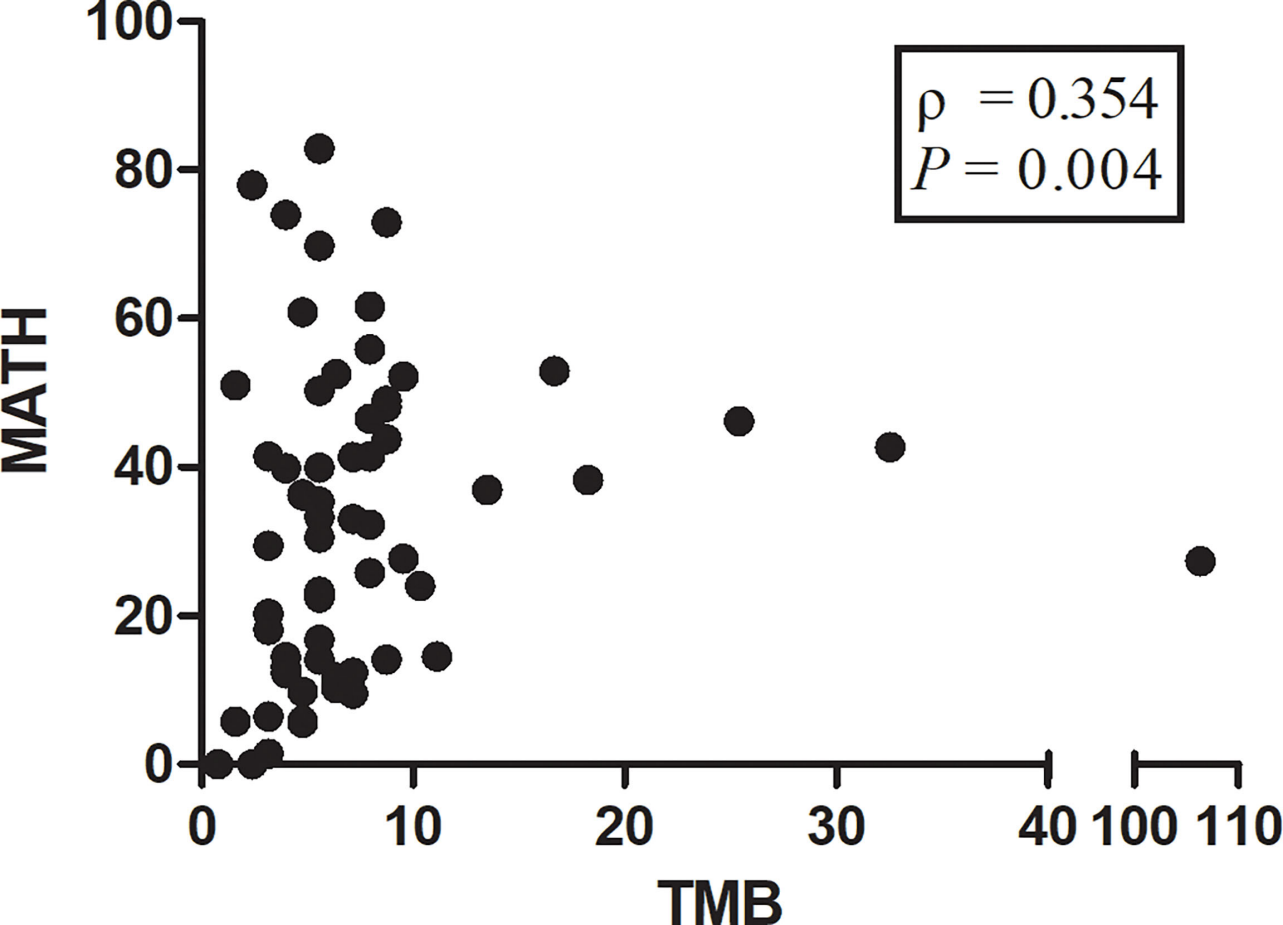
Scatterplot showing correlation between mutant-allele tumor heterogeneity (MATH) score and tumor mutational burden (TMB) score. The ρ value represents Spearman’s rank correlation. Spearman ρ = 0.354; P = 0.004.

We observed a significant correlation between TMB and MATH (Spearman ρ = 0.354; *P* = 0.004; **Figure 6**). No significant correlation was found between PD-L1 expression on tumor cells and TMB (Spearman ρ = -0.179; *P* = 0.161) or PD-L1 expression and MATH (Spearman ρ = -0.208; *P* = 0.101). Likewise, TMB and MATH had no significant correlation with PD-L1 expression on tumor infiltrating immune cells (Spearman ρ = 0.041, *P* = 0.752; Spearman ρ = -0.010, *P* = 0.937; respectively).

### Survival

**Figure 7:**
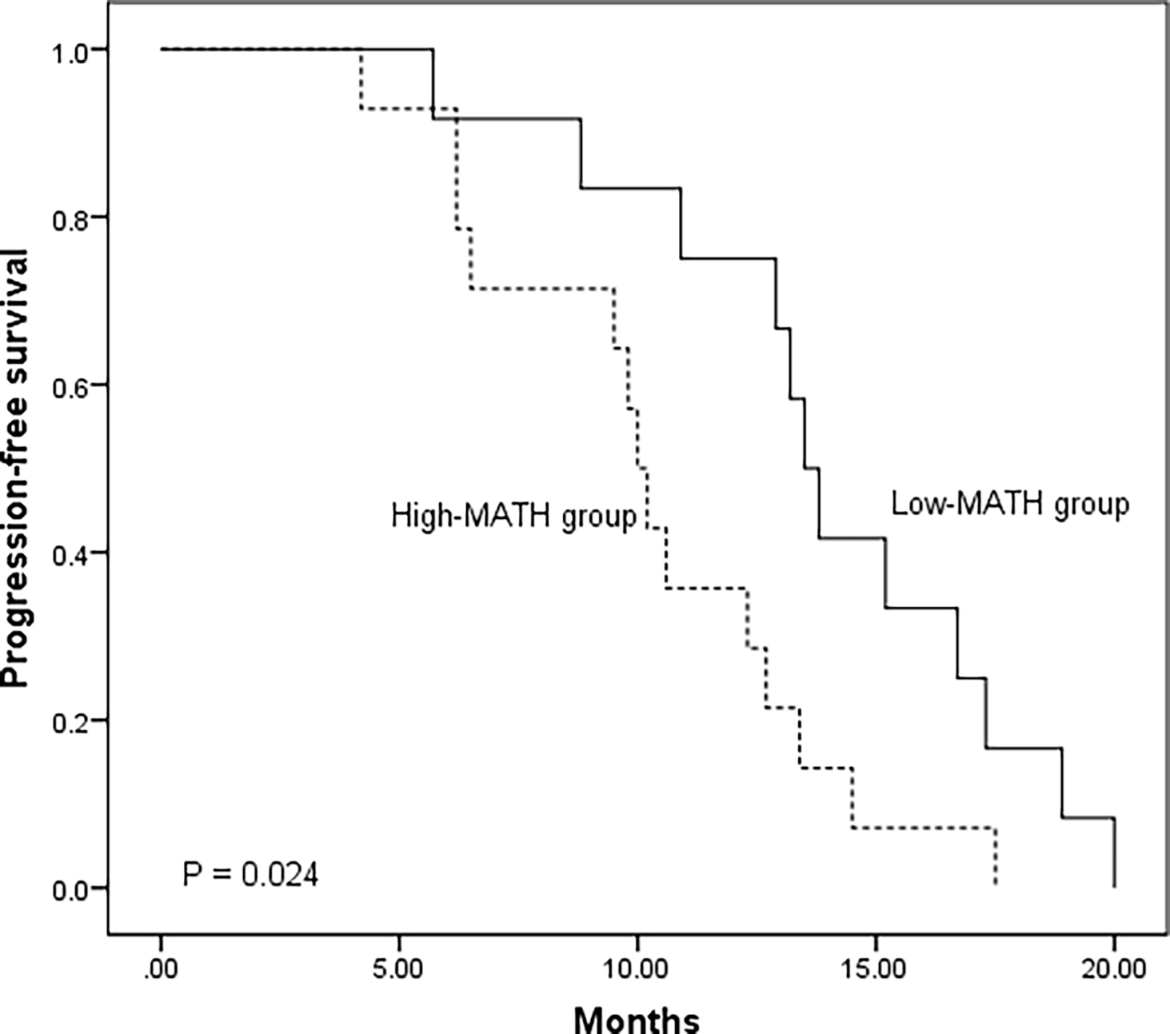
Kaplan–Meier analysis of progression-free survival in 26 *EGFR* mutated lung adenocarcinoma patients according to mutant-allele tumor heterogeneity (MATH) score (Low-MATH group vs. High-MATH group). The Low-MATH group demonstrated a higher objective response rate (ORR) than High-MATH group (75% vs. 21%, P = 0.016). The PFS was significantly longer among patients in Low-MATH group than among those in High-MATH group (median PFS: 13.7 months vs. 10.1 months; P = 0.024).

In our case series, 32 patients harbored *EGFR* mutation, among which 26 patients were treated with first generation *EGFR* TKI as single-agent therapy. We classified the 26 patients into two groups according to the median of MATH score: 14 patients with high MATH (High-MATH group) and 12 patients with low MATH (Low-MATH group). Treatment response and progression-free survival (PFS) of the 26 patients were shown in **Supplementary Table 2**. The Low-MATH group demonstrated a higher objective response rate (ORR) than High-MATH group (75% vs. 21%, *P* = 0.016). The PFS was significantly longer among patients in Low-MATH group than among those in High-MATH group (median PFS: 13.7 months vs. 10.1 months; *P* = 0.024; **Figure 7**).

## Discussion

In the study, we used MATH score to measure ITH and explored the association of MATH with clinical and molecular features in patients with advanced lung adenocarcinoma. Our data revealed that patients with stage IV tended to have a higher MATH than that with stage III. And our study had demonstrated that Low-MATH patients tended to had a higher ORR and better PFS. Moreover, we found *EGFR* mutation and disruptive *TP53* mutation were significantly associated with high level of MATH. In addition, a positive correlation between MATH and TMB was observed, suggesting its role in patients’ survival prediction. To the best of our knowledge, this is the first study focusing clinical and molecular relevance of MATH in advanced lung adenocarcinoma.

In terms of clinical predictors, we found that patients with more advanced stage and more distant metastases tended to have a higher MATH score. This finding has been validated in colorectal cancer in other work (12, 13). We also found that the presence of driver mutation in *EGFR* was associated with higher MATH score, which is consistent with a previous study based on multi-region sequencing showing high intra-tumor heterogeneity in *EGFR*-mutant lung adenocarcinoma (20). Mutations in *EGFR* and early *TP53* mutations involve in deregulating the cell cycle, evading cell death and facilitating tolerance of pervasive whole-genome doubling (WGD) and chromosomal instability (CIN), which results in early clonal selection with subsequent high intra-tumor heterogeneity. However, the results of aforementioned studies revealed low ITH in lung adenocarcinoma (8, 21). The reasons for the discordance cannot be fully elucidated and further studies are needed to validate the ITH in *EGFR*-mutant lung adenocarcinoma.

TP53 plays a vital role in activation of DNA repair pathway and regulation of apoptosis, which suggests that *TP53* mutation may contribute to instability of genome and increased intratumor genetic heterogeneity. *TP53* mutation has been reported to correlated with higher MATH in colorectal cancer and breast cancer (13, 14). Mroz EA et al. classified *TP53* mutations into disruptive and nondisruptive mutations and revealed that disruptive mutations in *TP53* were exclusively related to higher MATH in head and neck squamous cell carcinoma (10, 11). Consistent with the previous study, we found that higher MATH was specifically associated with disruptive but not with nondisruptive *TP53* mutations in lung adenocarcinoma. Some of the p53 protein functional properties can be retained in nondisruptive mutations, but disruptive mutations always lead to a complete loss of p53’s normal functions, which indicated that disruptive *TP53* mutations may have much more adverse impact on DNA repair and apoptosis regulation than nondisruptive *TP53* mutations, and thus cause higher intratumor genetic heterogeneity. We further investigated the correlation between mutations in DNA repair genetic pathways and MATH. Our results demonstrated that mutations in DNA repair pathways, especially mismatch repair (MMR) pathway and base excision repair (BER) pathway, predicted higher MATH scores, which further corroborated the impact of deficiencies in DNA-damage response on ITH.

TMB and PD-L1 expression have emerged as important biomarkers to predict immunotherapy response in advanced NSCLC (22, 23), whereas the association of MATH with TMB and PD-L1 expression remain unclear. A recent study reported that ITH had an impact on TMB estimation—ITH-high patients had significantly higher TMB compared with ITH-low patients (*P* < 0.001) (24). Similar to the previous report, a positive correlation between MATH and TMB was found in the present study. According to our results, the mechanism can be explained as that high MATH is closely related to deficiencies in DNA-damage response and repair, which contributes to high TMB (25, 26). In addition, MATH has no significant correlation with PD-L1 expression on tumor cells and tumor infiltrating immune cells. Based on the above, high MATH score may serve as a predictive biomarker independent of PD-L1 expression for the efficacy of immunotherapy. Due to lack of clinical immunotherapy outcomes, future studies are needed to confirm this hypothesis. And some other IHC markers will be identified to explore the relation in our future research.

The present study also has several limitations. First, it was a retrospective study and therefore selection bias was inevitable. Second, there is no standard cutoff for MATH and we stratified MATH using the median value in the present study. Third, due to a relatively small cohort, the results cannot be regarded as definitive.

## Conclusions

In summary, we used MATH to measure ITH and explored its association with clinical and molecular features in patients with advanced lung adenocarcinoma. The results of the present study indicated that MATH was associated with *EGFR*, *TP53* status and mutations of DNA repair pathway. In addition, MATH was found to have a positive correlation with TMB.

## Data Availability Statement

The datasets presented in this study can be found in online repositories. The names of the repository/repositories and accession number(s) can be found in the following: Genome Sequence Archive for Human (GSA-Human), as a part of GSA in the National Genomics Data Center - https://ngdc.cncb.ac.cn/gsa-human/, HRA002592.

## Ethics Statement

The studies involving human participants were reviewed and approved by institutional review board and ethics committee of Cancer Hospital, Chinese Academy of Medical Science. The patients/participants provided their written informed consent to participate in this study.

## Author Contributions

PS, XW, and WL analyzed clinical data and wrote the manuscript. LG provided the clinical samples and data. JY has been crucial in designing the work and supervising the draft revision. All authors read, revised and approved the final manuscript.

## Funding

Beijing Nova Program of Science and Technology (Z191100001119095), CAMS Innovation Fund for Medical Sciences (CIFMS) 2021-I2M-1-066, Beijing Hope Run Special Fund of Cancer Foundation of China (LC2019L04).

## Conflict of Interest

The authors declare that the research was conducted in the absence of any commercial or financial relationships that could be construed as a potential conflict of interest.

## Publisher’s Note

All claims expressed in this article are solely those of the authors and do not necessarily represent those of their affiliated organizations, or those of the publisher, the editors and the reviewers. Any product that may be evaluated in this article, or claim that may be made by its manufacturer, is not guaranteed or endorsed by the publisher.

## Abbreviations

ITH, Intra-tumor heterogeneity; MATH, mutant-allele tumor heterogeneity; NGS, next-generation sequencing; TMB, tumor mutational burden; EGFR, epidermal growth factor receptor; HR, homologous recombination; MMR, mismatch repair; BER, base excision repair; NER, nucleotide excision repair; NHEJ, nonhomologous end joining; TC, tumor cells; IC, tumor-infiltrating immune cells; PD-L1, programmed death ligand 1.
