# Supplementary material for: Mutant-Allele Tumor Heterogeneity, a Favorable Biomarker to Assess Intra-Tumor Heterogeneity, in Advanced Lung Adenocarcinoma
Source: Front Oncol. 2022 Jul 1;12:888951. doi: 10.3389/fonc.2022.888951 (PMC9286753; doi:10.3389/fonc.2022.888951)
Supplement: Supplementary file 2 [file Table_2.docx]

Supplementary Table 2. Tumor response with high MATH versus low MATH in patients with advanced lung adenocarcinoma.

| Case No. | EGFR mutation | EGFR TKI | MATH | Response | PFS (months) |
| --- | --- | --- | --- | --- | --- |
| 1 | Exon 21 L858R | Icotinib | Low | PR | 5.7 |
| 2 | Exon 19 del | Gefitinib | Low | PR | 8.8 |
| 3 | Exon 19 del | Gefitinib | Low | SD | 10.9 |
| 4 | Exon 19 del | Gefitinib | High | SD | 4.2 |
| 5 | Exon 21 L858R | Gefitinib | Low | PR | 12.9 |
| 6 | Exon 19 del | Gefitinib | Low | PR | 13.2 |
| 7 | Exon 21 L858R | Icotinib | High | SD | 6.2 |
| 8 | Exon 21 L858R | Icotinib | High | PD | 6.2 |
| 9 | Exon 21 L858R | Icotinib | High | SD | 6.5 |
| 10 | Exon 19 del | Icotinib | Low | PR | 13.8 |
| 11 | Exon 19 del | Icotinib | High | SD | 9.5 |
| 12 | Exon 21 L858R | Gefitinib | High | SD | 9.8 |
| 13 | Exon 19 del | Icotinib | High | SD | 10 |
| 14 | Exon 21 L858R | Icotinib | Low | PR | 13.5 |
| 15 | Exon 19 del | Icotinib | High | PR | 10.2 |
| 16 | Exon 15 G588S | Afatinib | High | PD | 10.6 |
| 17 | Exon 19 del | Gefitinib | Low | PD | 15.2 |
| 18 | Exon 19 del | Gefitinib | High | PR | 12.3 |
| 19 | Exon 21 L858R | Icotinib | High | PD | 12.7 |
| 20 | Exon 19 del | Icotinib | Low | SD | 16.7 |
| 21 | Exon 21 L858R | Icotinib | High | PR | 13.4 |
| 22 | Exon 21 L858R | Gefitinib | High | PD | 14.5 |
| 23 | Exon 21 L858R | Gefitinib | Low | PR | 17.3 |
| 24 | Exon 19 del | Gefitinib | Low | PR | 18.9 |
| 25 | Exon 19 del | Icotinib | Low | PR | 20 |
| 26 | Exon 19 del | Icotinib | High | SD | 17.5 |

Abbreviations: MATH, Mutant-allele tumor heterogeneity; PR, partial response; SD, stable disease; PD, progressive disease; PFS: progression-free survival; EGFR, epidermal growth factor receptor; TKI, tyrosine kinase inhibitor.
